# Storytelling in Motion: Effects of a Narrative-Based Outdoor Motor Intervention on Motor Competence and Inhibitory Control in Preschool Children—A Quasi-Experimental Study

**DOI:** 10.3390/children13060718

**Published:** 2026-05-22

**Authors:** Donatella Di Corrado, Maria Chiara Parisi, Matteo Pacifico Mancini, Patrizia Tortella

**Affiliations:** Department of Human and Social Sciences, Kore University of Enna, 94100 Enna, Italy; mariachiara.parisi@unikore.it (M.C.P.); matteopacifico.mancini@unikorestudent.it (M.P.M.); patrizia.tortella@unikore.it (P.T.)

**Keywords:** early childhood, motor competence, inhibitory control, storytelling, physical activity, preschool education

## Abstract

**Highlights:**

**What are the main findings?**

- Preschool children participating in narrative-based outdoor activities showed greater improvements in balance and fine motor performance.
- Free outdoor play was associated with greater improvements in coordination and upper-body strength tasks.
- No significant differences between groups were observed for inhibitory control.

**What are the implications of the main findings?**

- Outdoor motor activities may support different aspects of motor development depending on the instructional approach used.
- Narrative-based movement activities may promote children’s engagement and attention during motor tasks.
- Further randomized studies are needed to clarify the effects of instructional strategies and outdoor environments on child development.

**Abstract:**

**Background:** Promoting physical activity in early childhood is essential for supporting motor, cognitive, and socio-emotional development. Outdoor environments rich in natural stimuli may further enhance these benefits. Recent approaches suggest that integrating movement with narrative contexts may provide additional developmental opportunities by engaging cognitive and affective processes. This study examined the associations between three outdoor motor activity approaches—Storytelling in Motion, Free Play, and Traditional Motor Instruction—and motor competence and inhibitory control in preschool children. **Methods:** Eighty-seven preschool children (M_age = 5.32 ± 0.60 years) participated in a quasi-experimental pretest–posttest study conducted in outdoor educational settings in Northern Italy, including a natural environment, a structured playground, and a school courtyard. Participants were assigned at the class level to three groups of unequal size (Storytelling in Motion n = 36, Free Play n = 22, Traditional Motor Instruction n = 29). All groups completed ten weekly sessions lasting approximately 60 min. Motor competence was assessed using selected tasks derived from the Test of Motor Competence and the Movement Assessment Battery for Children-2, while inhibitory control was evaluated using the Day/Night Test. **Results:** Significant Time × Group interactions were observed for several outcomes. The Storytelling in Motion group showed numerically greater improvements at a descriptive level in dynamic balance (Heel-to-Toe Walking: *p* < 0.001, η^2^p = 0.229) and fine motor control (Bicycle Trail: *p* < 0.001, η^2^p = 0.194) compared to the other groups. The Free Play group showed greater improvements in coordination-related tasks and upper-body strength. No significant differences between groups were observed for inhibitory control. These differences remained significant after adjustment but should be interpreted cautiously due to the non-randomized design. Accordingly, these findings should be considered preliminary and hypothesis-generating (ANCOVA, *p* < 0.05). **Conclusions:** Narrative-based outdoor motor activities may represent a potentially relevant approach; however, no firm conclusions can be drawn from the present design. Given the quasi-experimental nature of the study and the contextual differences between intervention settings, the findings should be interpreted with caution. Future research using randomized controlled designs and standardized environments is needed to clarify the independent and combined effects of instructional and environmental factors.

## 1. Introduction

### 1.1. Motor Development and Outdoor Contexts

Promoting physical activity in early childhood is a key priority in both education and public health. A substantial body of evidence indicates that regular movement experiences during the preschool years support not only motor development, but also cognitive, emotional, and social functioning, while contributing to the prevention of noncommunicable diseases such as obesity and metabolic disorders [1,2]. Accordingly, the World Health Organization recommends that preschool children engage in at least 180 min of daily physical activity, including a minimum of 60 min of moderate-to-vigorous intensity activity, alongside adequate sleep and limited screen exposure [3,4]. Within this framework, early childhood education settings play a crucial role in fostering active lifestyles.

In preschool contexts, motor activity should not be viewed solely as an opportunity for play, but rather as a fundamental educational tool that contributes to children’s overall development. According to Maffeis [5,6] and Maffeis et al. [7], the prevention of childhood obesity requires early and multidimensional interventions based on the promotion of physical activity, the reduction in sedentary behaviors, and the adoption of healthy lifestyles from the earliest years of life. Early childhood represents a sensitive period for the acquisition of motor competence and the development of executive functions, including inhibitory control, working memory, and cognitive flexibility [8,9]. These abilities are closely associated with later academic achievement, self-regulation, and long-term health outcomes. In particular, motor competence has been identified as a key determinant of positive developmental trajectories, as it is linked to higher levels of physical activity, better physical fitness, and improved psychosocial well-being [10].

From a theoretical perspective, the framework of embodied cognition suggests that cognitive processes are grounded in sensorimotor experiences and emerge through active interaction with the environment. Movement, therefore, plays a central role not only in physical development, but also in shaping attention, action planning, and self-regulation.

In line with this perspective, previous research has shown that motor programs characterized by high levels of cognitive engagement—such as those involving coordination, problem solving, and adaptation to changing environmental demands—can positively influence inhibitory control, even in the absence of substantial changes in physical fitness [11]. A recent systematic review further highlights that physical education programs in preschool can improve children’s physical activity levels, motor competence, cognitive abilities, and social skills, particularly when they are structured, continuous, and diversified [12].

In addition to the nature of the activities, the context in which movement occurs appears to be particularly relevant. Pesce et al. [13] highlighted that motor experiences based on exploration, variability, adaptation, and active participation simultaneously promote motor and cognitive development. Outdoor and green environments may provide additional benefits because they offer more varied opportunities for movement, exploration, and sensory stimulation than indoor settings or traditional playgrounds. Compared to indoor environments, natural settings often include uneven surfaces, slopes, obstacles, and open spaces, which may challenge balance, coordination, and motor planning [14]. Exposure to green spaces has also been associated with improvements in attention, behavioral regulation, and cognitive development in children [15,16]. These findings suggest that outdoor environments, rich in stimuli and characterized by unpredictable situations, may be linked to developmental benefits, particularly through increased opportunities for variability, exploration, and sensorimotor stimulation [17,18].

In this regard, research showed that structured activities carried out in specifically designed playgrounds can significantly improve preschool children’s motor development, particularly in balance, coordination, object control skills, and inhibitory control [19,20,21,22].

### 1.2. Pedagogical Approaches in Early Childhood

From an educational perspective, preschool pedagogies often oscillate between unstructured free play and traditional motor instruction. Free play promotes autonomy, creativity, and social interaction, as highlighted in previous research on early childhood education [23,24]. It provides children with opportunities to explore their environment, make independent decisions, and engage in self-directed activities, which can support the development of problem-solving skills and intrinsic motivation [25,26]. Moreover, the open-ended nature of free play allows children to engage in repeated practice of motor skills within meaningful contexts, potentially contributing to both motor and cognitive development [27,28]. In contrast, traditional motor instruction offers more structured practice but may limit emotional, imaginative, and motivational involvement [29].

It is important to clarify that Traditional Motor Instruction in preschool settings is not necessarily devoid of engagement or meaningful context. In many cases, structured motor activities include elements of play, interaction, and teacher support that promote motivation and participation [30]. In the present study, the distinction between Traditional Motor Instruction and the other conditions refers primarily to the level of structure and the absence of an explicit narrative framework, rather than to differences in engagement or educational value.

### 1.3. Narrative-Based Movement and Cognitive Processes

In recent years, increasing attention has been devoted to integrated approaches that combine movement with cognitive and affective engagement. Among these, narrative-based motor activities—often referred to as Storytelling in Motion—have been proposed as a promising educational strategy [31]. In this approach, motor tasks are embedded within meaningful narrative contexts, allowing children to engage in movement while following a story, solving problems, and adopting goal-directed behaviors [32].

This integration may be associated with increased motivation, sustained attention, and emotional involvement, as children participate in meaningful and goal-oriented activities within a structured narrative framework. Narrative contexts can provide purpose and coherence to motor actions, potentially enhancing engagement and encouraging active participation [33]. Moreover, the integration of cognitive, affective, and motor demands may promote deeper involvement in the activity, thereby amplifying the developmental benefits of physical activity. For example, Duncan et al. [34] reported that combined movement and storytelling interventions are associated with greater improvements in motor and language competence compared to activities based exclusively on movement or storytelling. Similarly, Eyre et al. [35] found improvements in fundamental motor skills—such as running, jumping, throwing, and catching—particularly among children with fewer opportunities for physical activity. In addition, Cunningham et al. [36], through the MAST program, demonstrated that integrated movement and storytelling activities can be effectively implemented by teachers and may support both language and motor development. These interpretations are consistent with previous research suggesting that cognitively engaging and context-rich motor activities can support both motivational processes and attentional regulation in early childhood [37].

Narrative-based movement activities may influence inhibitory control by requiring children to follow rules, suppress automatic responses, and adapt their behavior within a structured storyline, thereby increasing cognitive demands during motor execution. In such contexts, children must monitor task goals, maintain relevant information, and inhibit prepotent responses, processes that are central to executive functioning. This integration of cognitive and motor demands may support the development of executive processes, particularly response inhibition and attentional control [38,39].

Furthermore, motor competence may be associated with narrative comprehension and broader cognitive development, potentially through mechanisms involving executive functions such as working memory and inhibitory control [40]. Children with higher motor competence may be better able to engage in complex, goal-directed activities that require the integration of action and cognition, including the ability to follow and interpret narrative sequences [41]. From this perspective, motor and cognitive processes may interact dynamically, as effective movement execution relies on planning, monitoring, and adaptation [42]. These interactions may contribute not only to motor skill acquisition but also to language-related processes, such as narrative understanding and organization, thereby supporting more integrated developmental outcomes [43].

### 1.4. Study Rationale and Aims

Despite these promising indications, relatively few studies have directly compared narrative-based motor interventions with more traditional approaches in preschool settings, particularly within outdoor environments. Furthermore, it remains unclear whether different pedagogical models are associated with distinct effects across specific domains of motor competence and aspects of executive functioning.

It is important to note that, in real educational contexts, different pedagogical approaches are often implemented within distinct physical environments. In the present study, the three intervention conditions were conducted in settings with different environmental characteristics (i.e., a natural outdoor environment, a structured playground, and a school courtyard). Therefore, the comparison between groups reflects not only differences in instructional approach, but also differences in environmental affordances for movement. As a result, the study does not aim to isolate the independent effect of the pedagogical model, but rather to examine how different combinations of instructional strategies and environmental contexts are associated with motor and cognitive outcomes in preschool children.

In light of these considerations, the present study aimed to compare the effects of three outdoor motor activity approaches—Storytelling in Motion, Free Play, and Traditional Motor Instruction—on motor competence and inhibitory control in preschool children. Particular attention was devoted to key domains of motor skills, including object control, locomotor abilities, balance, physical fitness, and coordination, as well as to inhibitory control. A quasi-experimental pretest–posttest design was adopted, with each group participating in ten weekly sessions conducted in outdoor settings. The Storytelling in Motion group carried out activities in a historic garden, the Free Play group operated in a structured playground, and the Traditional Motor Instruction group carried out its activities in the courtyard of a preschool. The three contexts presented comparable characteristics in terms of size, geographical location, and climatic conditions: all activities took place in Northern Italy, during the same period of the year, in springtime, and involved children from families with a medium socioeconomic background. Since motor activities were part of the regular preschool curriculum, all groups pursued the developmental goals established by the Italian National Curriculum Guidelines [44]. In Italy, each school has a certain degree of teaching autonomy and may adopt different educational strategies to achieve the same learning objectives. Given the characteristics of the study design, it was hypothesized that the three intervention conditions would be associated with different patterns of change across motor and cognitive outcomes. Specifically, Storytelling in Motion was expected to be associated with greater improvements in balance, fine motor control, and inhibitory processes, whereas Free Play was expected to be associated with improvements in coordination and strength-related tasks. Traditional Motor Instruction was expected to show more moderate changes.

## 2. Materials and Methods

### 2.1. Study Design

This study employed a quasi-experimental, non-randomized pretest–posttest design to examine the associations between three outdoor motor activity approaches and motor competence and inhibitory control in preschool children. Participants were assigned at the class level to one of three intervention conditions—Storytelling in Motion, Free Play, or Traditional Motor Instruction—based on their existing school organization. As a result, random allocation was not feasible.

This design reflects real educational practice but introduces potential selection bias and limits causal inference. Accordingly, the findings should be interpreted as exploratory. All groups were assessed at two time points (pre-intervention and post-intervention), allowing the evaluation of within-group changes over time and between-group differences in developmental trajectories.

### 2.2. Participants

The sample comprised 87 preschool children (Mage = 5.32 years, SD = 0.60) attending three kindergartens in Northern Italy with comparable sociocultural and socioeconomic characteristics. Participants were distributed into three naturally occurring class groups: Storytelling in Motion (n = 36), Free Play (n = 22), and Traditional Motor Instruction (n = 29).

Children were eligible if they were enrolled in the final year of kindergarten (typically aged 5–6 years), regularly attending school, and able to participate in standard motor activities without restrictions. Exclusion criteria included diagnosed neurological, developmental, or physical conditions affecting motor performance, as well as incomplete data at pretest or posttest.

**Figure 1:**
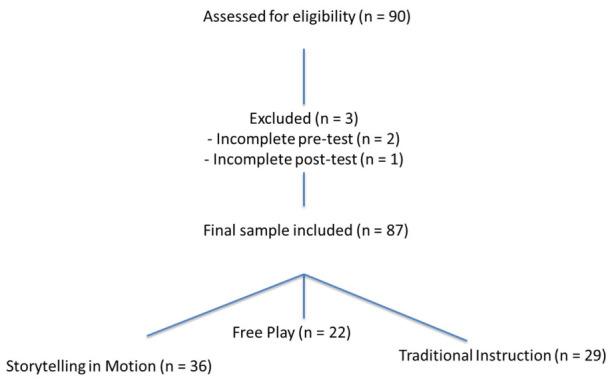
Participant flow diagram.

A total of 90 children were initially assessed for eligibility. Of these, 2 were excluded due to incomplete pre-test data and 1 due to incomplete post-test data. The final sample consisted of 87 children. The flow of participants from eligibility assessment to final inclusion is illustrated in Figure 1.

The study was conducted in accordance with the Declaration of Helsinki and approved by the local Ethics Committee (approval number: 612/2024/MEDF-02/16; approval date: 11 November 2024). Written informed consent was obtained from parents or legal guardians prior to participation.

### 2.3. Procedure

All participants completed a pretest–posttest assessment protocol. Baseline assessments were conducted prior to the intervention, and identical procedures were repeated immediately after the intervention period.

Each group participated in 10 outdoor sessions, conducted once per week over a period of approximately ten weeks. Each session lasted approximately 60 min and was led by instructors with degrees in exercise science who had received specific training on the intervention protocols.

To ensure consistency across conditions:

- All sessions were conducted during morning hours (between 9:00 a.m. and 12:00 p.m.);
- Similar developmental objectives were targeted across groups (motor competence and inhibitory control);
- Instructors followed predefined activity plans;
- Intervention fidelity was monitored through periodic checks to verify adherence to the planned protocols.

Preschool teachers were present during sessions for supervision and safety but did not actively lead the activities.

It should be noted that the three intervention conditions were implemented in different physical environments, which varied in terms of surface characteristics, structural complexity, and availability of equipment. Specifically, the Storytelling in Motion condition took place in a natural outdoor setting with uneven terrain and natural obstacles, the Free Play condition in a structured playground with fixed equipment, and the Traditional Motor Instruction condition in a school courtyard. As a result, the intervention conditions differed not only in pedagogical approach but also in environmental affordances for movement.

### 2.4. Intervention Conditions

The three intervention conditions followed a structured session plan designed to reflect typical educational practices, while differing in instructional approach and level of guidance.

#### 2.4.1. Storytelling in Motion

In the Storytelling in Motion condition, motor activities were embedded within structured narrative scenarios. Each session represented part of an ongoing story, divided into sequential episodes through which children progressed over the intervention period. Children engaged in narrative-based activities involving exploration and goal-directed tasks (e.g., overcoming obstacles, navigating environments, and completing missions). This approach was designed to promote imagination and emotional engagement, sustained attention, goal-directed behavior, and the integration of cognitive and motor processes. Activities were conducted in a historic garden characterized by natural elements such as uneven terrain, slopes, trees, stones, and open spaces. No fixed playground equipment was used; instead, children interacted with natural features and simple materials provided by the instructors. The intervention was delivered according to predefined narrative scenarios, and general adherence to the planned activities was monitored throughout the intervention period. However, no standardized measures of children’s engagement with the narrative (e.g., attention, recall, or motivation) or formal assessments of fidelity of narrative delivery were collected. Importantly, specific cognitive demands were embedded within the narrative structure. Children were required to follow rules defined by the storyline (e.g., stopping when a signal was given or changing direction based on cues), inhibit automatic responses (e.g., resisting the impulse to move before receiving instructions), and maintain task goals across sequential activities. For example, children participated in scenarios such as crossing an imaginary river by stepping only on designated safe areas, navigating obstacles to complete a mission, or collecting objects in a specific order while adhering to story-based rules. These activities required continuous adaptation of behavior, attention to instructions, and goal-directed action, thereby increasing cognitive engagement during motor execution.

#### 2.4.2. Free Play

In the Free Play condition, children engaged in spontaneous, self-directed outdoor activities with minimal adult guidance. Sessions took place in a structured playground equipped with fixed play structures, such as climbing frames, slides, and open spaces. Children were free to choose their activities and move autonomously within the environment. Adult involvement was limited to ensuring safety and responding to children’s requests when necessary. No structured tasks, explicit instructions, or narrative elements were introduced. This condition emphasized autonomy, exploration, and self-initiated practice, allowing children to engage in repeated and self-selected motor activities. Such conditions may promote motor learning through repeated practice and spontaneous exploration of environmental affordances.

#### 2.4.3. Traditional Motor Instruction

The Traditional Motor Instruction condition consisted of instructor-led structured motor activities, typical of preschool physical education programs. Sessions were conducted in an outdoor school courtyard and included organized movement games, motor circuits targeting coordination, balance, and fundamental motor skills, as well as repetitive practice of specific motor tasks. Activities were guided and structured, with clearly defined instructions and objectives. Unlike the Storytelling in Motion condition, activities were not embedded within a narrative context. In contrast to the Free Play condition, opportunities for spontaneous and self-directed activity were limited, ensuring a clear distinction between the pedagogical approaches. It should be noted that the level of adult involvement differed across conditions. Both the Storytelling in Motion and Traditional Motor Instruction conditions involved instructor-led activities with structured guidance, whereas the Free Play condition was characterized by minimal adult intervention. These differences in adult attention, verbal instruction, and feedback may have influenced children’s engagement and performance and should be considered as a potential confounding factor.

### 2.5. Measures

Analyses were conducted at the level of individual task outcomes rather than aggregated subscale or total scores, as the tasks were adapted and not administered as complete standardized batteries. The outcome variables (Supplementary File S1) included in the analyses were: Heel-to-Toe Walking, Walking/Running in Slope, Bicycle Trail, Building Bricks, Placing Bricks (left and right hand), Catching Beanbag, Medicine Ball, and Day/Night Test. Although multiple tasks were described within each assessment domain, only selected task-level outcomes were included in the statistical analyses in order to focus on representative indicators of motor competence and cognitive performance.

### 2.6. Assessment of Motor Competence

Motor competence was assessed both before and after the intervention period, following completion of all activities in the three groups. Assessment procedures were based on selected tasks derived from widely used and validated instruments, including the Test of Motor Competence (TMC), the Movement Assessment Battery for Children—Second Edition (MABC-2), and gross motor performance tests. Rather than administering the full standardized batteries, specific tasks were selected and adapted for implementation in an outdoor educational context. These tasks were chosen to represent key domains of motor competence, including balance, manual dexterity, coordination, and object control. For time-based tasks (e.g., Building Bricks, Heel-to-Toe Walking), lower scores indicate better performance, reflecting shorter completion times. For accuracy-based tasks, lower scores indicate fewer errors, whereas for strength- or distance-based tasks, higher scores indicate better performance.

### 2.7. Test of Motor Competence (TMC)

The TMC [45] includes four tasks designed to assess both fine and gross motor skills. Two tasks focus on manual dexterity, whereas the other two evaluate dynamic balance and locomotor abilities. Performance in each task is recorded as the time required to complete the activity, expressed in seconds.

The fine motor section included Placing Bricks (left and right hand) and Building Bricks (tower construction using bricks). The Platform Bricks tasks assessed manual dexterity through the timed placement of small objects onto designated targets (left- and right-hand conditions). These tasks required precision, coordination, and speed, and were used as indicators of fine motor control and visuomotor integration.

The gross motor section included Heel-to-Toe Walking and Walking/Running in Slope. Before each task, children were allowed one practice trial. Previous research has demonstrated satisfactory internal consistency, construct validity, and test–retest reliability for the TMC.

Heel-to-Toe Walking was used as an indicator of dynamic balance. Children were instructed to walk as quickly as possible along a 4.5 m straight line, placing the heel of each foot directly in front of the toes of the opposite foot at each step. The Walking/Running in Slope task was adapted from the Figure-of-Eight Test described by Johansson and Jarnlo [46], which assesses locomotor coordination, agility, and the ability to change direction. Children moved as quickly as possible in a figure-of-eight pattern around two markers positioned at different distances from the starting point, alternating direction around each marker before returning to the starting line.

### 2.8. Movement Assessment Battery for Children—Second Edition (MABC-2)

The MABC-2 [47] is designed to assess fine and gross motor coordination in children aged 3 to 16 years. In the present study, the version for children aged 3 to 6 years (Age Band 1) was used. The assessment included selected tasks from the domains of manual dexterity, object control skills, and balance.

#### 2.8.1. Manual Dexterity

Manual dexterity tasks included Posting Coins, Threading Beads, and Bicycle Trail. Lower scores indicated better performance, reflecting shorter completion times or fewer errors. Posting Coins required children to insert coins into a slot as quickly as possible, assessing speed, hand–eye coordination, and fine motor precision. Threading Beads evaluated bimanual coordination, motor planning, and visuomotor integration.

The Bicycle Trail task was a graphomotor tracing activity adapted from the manual dexterity domain of the MABC-2 (Age Band 1), in which children traced a predefined path while minimizing errors. This task was used as an indicator of fine motor control and visuomotor integration.

#### 2.8.2. Aiming and Catching

Object control skills were assessed through a beanbag catching task. Higher scores indicated better performance. The Catching a Beanbag task required children to catch a beanbag thrown toward them and was used as an indicator of hand–eye coordination, anticipation, timing, and gross motor control. In the present study, only the Catching a Beanbag task was included among the Aiming and Catching measures.

#### 2.8.3. Balance

Balance tasks included One-Leg Balance, Walking Heels Raised, and Jumping on Mats. Higher scores indicated better performance, reflecting longer duration, greater stability, or fewer errors. One-Leg Balance assessed static balance and postural control. Walking Heels Raised evaluated dynamic balance and coordination. Jumping on Mats required children to perform controlled jumps while maintaining stability and accurate landings.

#### 2.8.4. Gross Motor Performance

Additional gross motor tasks included the Standing Broad Jump [48] and the Medicine Ball throw. Higher scores indicated better performance. The Standing Broad Jump assessed lower-body explosive strength, while the Medicine Ball task evaluated upper-body explosive strength. Children performed two trials, and the best performance was recorded.

#### 2.8.5. Inhibitory Control

Inhibitory control was assessed using the Day/Night Test [49], a Stroop-like task widely used in preschool populations. This test specifically evaluates response inhibition, defined as the ability to suppress a dominant response and provide an alternative rule-based response.

Although inhibitory control is considered one component of executive functioning, the present study did not aim to assess executive functioning as a multidimensional construct. In the task, children were required to provide the opposite verbal response to visual stimuli (e.g., saying “night” when shown a picture of the sun and “day” when shown a picture of the moon), thereby inhibiting automatic responses. Successful performance required children to inhibit the prepotent response, maintain the rule in working memory, and apply it correctly. The outcome measure was the number of errors, with lower scores indicating better inhibitory control.

### 2.9. Statistical Analysis

Data were analyzed using IBM SPSS Statistics (Version 27; IBM Corp., Armonk, NY, USA). Descriptive statistics are reported as means and standard deviations (M ± SD). Preliminary analyses included checks for normality (Shapiro–Wilk test) and the identification of potential outliers. To evaluate intervention effects, mixed between–within subjects’ analyses of variance (mixed ANOVA) were conducted for each outcome variable, with Time (pretest vs. posttest) as the within-subject factor and Group (Storytelling in Motion, Free Play, Traditional Motor Instruction) as the between-subject factor. The main effect of Time assessed overall changes across participants, while the Group × Time interaction examined differences in change patterns between groups.

To examine baseline differences, one-way ANOVA was conducted for all outcome variables at pre-test. In addition, analyses of covariance (ANCOVA) were performed using post-test scores as dependent variables and corresponding pre-test scores as covariates, in order to control for baseline variability across groups. Effect sizes were calculated using partial eta squared (η^2^p) and interpreted as small (≈0.01), medium (≈0.06), and large (≈0.14). Statistical significance was set at *p* < 0.05.

Given the number of outcome variables analyzed and the absence of correction for multiple comparisons, the results should be interpreted with caution due to the increased risk of Type I error. Accordingly, statistically significant findings should be considered preliminary and hypothesis-generating rather than confirmatory. No formal correction for multiple comparisons was applied, as the analyses were exploratory in nature.

## 3. Results

Descriptive statistics for the anthropometric characteristics of the participants are presented in Table 1. No statistically significant differences were observed between the three groups in age, height, weight, or waist circumference (all *p* > 0.05), indicating that the groups were comparable with respect to basic physical characteristics at baseline.

However, as expected in a non-randomized design, some variability was observed in baseline performance across specific motor and cognitive measures. These differences were taken into account in the interpretation of the results.

A series of mixed between–within subjects’ analyses of variance (mixed ANOVA) were conducted to examine the effects of Time (pretest vs. posttest), Group (Storytelling in Motion, Free Play, Traditional Motor Instruction), and their interaction on motor competence and inhibitory control outcomes. Full descriptive statistics provided in Table 2A; only the main findings are reported in Table 2B.

A significant Time × Group interaction was observed for the Heel-to-Toe Walking test (F(2, 84) = 12.45, *p* < 0.001, η^2^p = 0.229), along with a significant main effect of Time (F(1, 84) = 35.52, *p* < 0.001, η^2^p = 0.216), indicating overall improvement across participants. The Storytelling in Motion group showed the largest reduction in completion time (from M = 49.98 to M = 24.09), reflecting a marked improvement in dynamic balance. The Free Play group also improved (from M = 39.54 to M = 31.84), although to a lesser extent. In contrast, the Traditional Motor Instruction group showed minimal change over time.

For the Bicycle Trail task, a significant Time × Group interaction was found (F(2, 84) = 10.59, *p* < 0.001, η^2^p = 0.194), while the main effect of Time was not significant. The Storytelling in Motion group demonstrated a reduction in errors, whereas the Free Play group remained relatively stable and the Traditional Motor Instruction group showed a slight increase in errors.

For the Building Bricks task, both a significant main effect of Time (*p* = 0.036) and a significant Time × Group interaction (*p* = 0.042) were observed. Given that this task is time-based, lower scores indicate better performance. The Storytelling in Motion group showed a slight improvement (reduction in completion time), whereas both the Free Play and Traditional Motor Instruction groups showed increases in completion time, indicating a worsening of performance, particularly in the Traditional group.

No significant effects were observed for the Placing Bricks (left hand) task, suggesting stable performance across time and groups. For the Placing Bricks (right hand) task, a significant Time × Group interaction was found (F(2, 84) = 7.24, *p* = 0.002, η^2^p = 0.145), along with a significant main effect of Group (*p* = 0.042, η^2^p = 0.074). Both the Storytelling in Motion and Free Play groups showed improvements (reduced completion times), whereas the Traditional Motor Instruction group showed a worsening of performance.

For the Walking/Running in Slope task, a significant main effect of Time was observed (F(1, 84) = 5.24, *p* = 0.008, η^2^p = 0.112), indicating overall improvement across participants. The interaction effect approached significance (*p* = 0.062, η^2^p = 0.089), suggesting a trend toward differential improvement between groups. The Free Play group showed the largest numerical improvement, followed by the Storytelling in Motion group, while the Traditional Motor Instruction group remained relatively stable.

For the Medicine Ball task, no significant Time × Group interaction was observed (*p* = 0.813), indicating that improvements were similar across groups. However, a significant main effect of Time was found (F(1, 84) = 13.13, *p* < 0.001, η^2^p = 0.216), suggesting that all participants improved their upper-body strength over the intervention period. Although all groups showed gains, the Free Play group demonstrated the largest numerical increase, followed by the Storytelling in Motion and Traditional Motor Instruction groups.

A significant main effect of Time was observed for the Day/Night Test (F(1,84) = 17.31, *p* < 0.001, η^2^p = 0.162), indicating overall improvement in inhibitory control across participants. However, the Time × Group interaction was not statistically significant (F(2,84) = 0.84, *p* = 0.425), suggesting that changes over time did not differ significantly between groups. Descriptively, the Storytelling in Motion group showed the largest numerical reduction in errors, followed by the Free Play and Traditional Motor Instruction groups. For the Catching Beanbag task, a significant main effect of Time was observed (F(1,84) = 8.03, *p* = 0.005, η^2^p = 0.045), indicating overall improvement across participants. A significant Time × Group interaction was also found (F(2,84) = 4.78, *p* = 0.010, η^2^p = 0.054), suggesting different patterns of change across groups. Descriptive data indicated greater improvements in the Free Play and Traditional Motor Instruction groups compared to the Storytelling in Motion group.

Pre–Post Changes across groups and best performing intervention by outcome variable are reported in Table 3. To avoid ambiguity, the direction of performance (i.e., whether lower or higher values indicate improvement) is explicitly specified for each outcome.

Given that lower scores indicate better performance, the Storytelling in Motion group showed a slight improvement (reduction in completion time), whereas both the Free Play and Traditional Motor Instruction groups showed increases in completion time, indicating a worsening of performance.

### ANCOVA Analyses Controlling for Baseline Differences

To account for baseline variability across groups, additional analyses of covariance (ANCOVA) were conducted for each outcome variable, using post-test scores as dependent variables and corresponding pre-test scores as covariates (Table 4).

A significant effect of Group was observed for Heel-to-Toe Walking (F(2,83) = 4.42, *p* = 0.015, η^2^p = 0.096) and Bicycle Trail (F(2,83) = 5.54, *p* = 0.006, η^2^p = 0.118), indicating that group differences remained statistically significant after adjustment, although this should be interpreted with caution given the non-randomized design and baseline differences. No significant group effects were found for Building Bricks (*p* = 0.329) or Day/Night Test (*p* = 0.806). Estimated marginal means are presented in Table 5.

Bonferroni-adjusted post hoc comparisons (Table 6) indicated statistically significant differences between groups. In particular, the Storytelling in Motion group showed lower adjusted scores than the Traditional Motor Instruction group for both Heel-to-Toe Walking (*p* = 0.009) and Bicycle Trail (*p* = 0.004), and lower scores than the Free Play group for Heel-to-Toe Walking (*p* = 0.041). However, these differences should be interpreted with caution, as they may reflect both improvement in the Storytelling in Motion group and worsening performance in other groups.

## 4. Discussion

The present study aimed to examine the associations between three outdoor motor activity approaches—Storytelling in Motion, Free Play, and Traditional Motor Instruction—and motor competence and inhibitory control (as a specific component within the broader domain of executive functioning) in preschool children. The findings are interpreted below by considering each activity condition in relation to the observed patterns of results, rather than focusing on individual test outcomes. Overall, the results suggest that the observed effects are domain-specific and dependent on the characteristics of the activity context, rather than reflecting a general advantage of a single approach [21].

### 4.1. Storytelling in Motion

The Storytelling in Motion condition was associated with more consistent improvements in balance and fine motor tasks. For example, children in this group showed the largest improvement in dynamic balance, as assessed by the Heel-to-Toe Walking task, and a slight improvement in fine motor performance in the Building Bricks task. However, these findings should be interpreted with caution. Improvements may be partly attributable to the characteristics of the natural outdoor environment, including uneven terrain, slopes, and variable surfaces, which are known to promote postural control, balance adaptation, and motor variability. Therefore, the observed effects cannot be attributed solely to the narrative-based component of the intervention.

At the same time, embedding motor challenges within a narrative context may have been associated with increased motivation and attentional engagement, potentially supporting more effective practice [50]. Narrative-based motor activities may influence inhibitory control through multiple complementary mechanisms. Within a structured storyline, children are required to follow rules, inhibit automatic responses, maintain task goals, and adapt their behavior to changing conditions. These processes increase cognitive demands during movement and may be associated with the development of executive functions. In particular, the narrative structure may be linked to sustained attention, while goal-directed tasks may support inhibitory control and response accuracy. These interpretations are consistent with embodied cognition frameworks and previous research on cognitively engaging motor activities [14,51].

### 4.2. Free Play

The Free Play condition was associated with more pronounced improvements in coordination-related tasks and upper-body strength. For example, children in this group showed favorable results in Placing Bricks and Walking/Running in Slope, as well as greater gains in strength-related tasks. These outcomes may reflect the opportunities for spontaneous exploration and repeated self-selected practice typical of free outdoor play [52]. However, these findings should also be interpreted with caution. The activities took place in a structured playground equipped with fixed elements such as climbing frames and slides, which may have provided repeated opportunities for climbing, jumping, and object manipulation. These environmental affordances are known to support the development of coordination and muscular strength. Therefore, the observed advantages may reflect not only the characteristics of free play as a pedagogical approach, but also the influence of the physical environment. As such, it is not possible to attribute these effects specifically to the Free Play condition alone.

### 4.3. Traditional Motor Instruction

The Traditional Motor Instruction condition showed more stable or limited changes across most outcomes. In some tasks, such as Building Bricks and Bicycle Trail, performance worsened over time, while in others improvements were minimal. These patterns may reflect differences in task structure, practice variability, and environmental constraints compared to the other conditions.

### 4.4. Cross-Condition Interpretation and Task-Specific Effects

With regard to strength, all groups improved similarly in the Medicine Ball task, suggesting that upper-body explosive strength may be associated with general maturation and regular participation in motor activities, regardless of the educational approach adopted. Some findings require careful interpretation. For example, in the Bicycle Trail task, the observed group differences appear to reflect not only improvement in the Storytelling in Motion condition, but also worsening performance in the Traditional Motor Instruction group. Therefore, these differences are more appropriately interpreted as reflecting divergence between groups, rather than a specific advantage of a single intervention.

Similarly, although differences were observed in the Medicine Ball task, comparable patterns were not found in the object control task included in the MABC-2. In the Catching Beanbag task, improvements were observed across all groups, with greater gains in the Free Play and Traditional Motor Instruction conditions compared to the Storytelling in Motion group. This pattern suggests that differences between groups may reflect task-specific demands and environmental affordances rather than a consistent advantage of a single approach. This discrepancy may be explained by differences in task demands. The Medicine Ball task primarily assesses upper-body explosive strength and gross motor coordination, whereas the Catching Beanbag task involves accuracy, timing, and perceptual–motor integration. Therefore, these results may reflect task-specific adaptations rather than a consistent effect across object control skills.

### 4.5. Inhibitory Control

All groups showed improvements in inhibitory control over time, with no significant differences between intervention conditions. Although the Storytelling in Motion group showed the largest numerical reduction in errors, this difference was not statistically significant and should therefore not be interpreted as evidence of a differential effect between groups. Accordingly, inhibitory control cannot be considered a differential outcome of the intervention conditions in the present study.

These findings suggest that participation in regular motor activity, regardless of instructional approach, may be associated with the development of inhibitory processes during early childhood [53]. However, the descriptive advantage observed in the narrative-based group may indicate that activities involving rule-following and behavioral regulation could provide additional cognitive stimulation. These interpretations remain tentative and should be considered with caution.

### 4.6. Adjusted Analyses and Overall Interpretation

In addition to the descriptive and mixed-model analyses, further insight into group differences was obtained through adjusted analyses. The inclusion of ANCOVA analyses allowed for a more rigorous evaluation of group differences by controlling for baseline variability across participants. These differences remained significant after adjustment but should be interpreted cautiously due to the non-randomized design. Accordingly, these findings should be considered preliminary and hypothesis-generating.

It is important to note that the present design does not allow the effect of storytelling to be isolated as an independent variable. The observed differences between groups may reflect the combined influence of multiple interacting factors, including environmental context, availability of equipment, and level of adult guidance, rather than the narrative component itself.

### 4.7. Limitations and Future Directions

Several limitations should be considered when interpreting the findings. First, the quasi-experimental design with non-randomized group allocation limits causal inference. Second, the interventions were implemented in different environmental contexts, making it difficult to disentangle instructional and environmental effects. Third, baseline differences between groups may have influenced the observed changes over time. In particular, the poorer initial performance of the Storytelling in Motion group in some outcomes may have allowed greater room for improvement, raising the possibility of regression to the mean. As a result, the observed differences may partly reflect initial group disparities rather than intervention-related effects. Fourth, the relatively short duration of the intervention may have limited the development of stable changes across all domains. Furthermore, in the absence of a no-intervention control group, it is not possible to determine whether observed changes reflect intervention-related effects or natural variation over time. Additionally, cognitive outcomes were assessed using a single measure of inhibitory control, which does not capture the full range of executive functions such as working memory and cognitive flexibility. Furthermore, the study did not include direct measures of children’s engagement with the narrative or of fidelity of implementation. As a result, it is not possible to determine whether the narrative component was consistently delivered or effectively experienced by participants, or whether it influenced behavior as intended. Therefore, any interpretation of the role of narrative-based activities should be considered tentative and speculative.

Moreover, the use of adapted tasks derived from standardized assessments may limit comparability with normative data. Finally, differences in the level of adult involvement across conditions may have influenced engagement and performance.

### 4.8. Strengths and Practical Implications

Despite these limitations, the study has several strengths, including its ecological validity, the comparison of multiple pedagogical approaches, and the integration of motor and cognitive outcomes in a real educational context. The findings suggest that combining structured activities, narrative engagement, and opportunities for autonomous exploration may be associated with different domains of motor and cognitive development. Future research should aim to employ randomized controlled designs, larger samples, and longer intervention periods, and to examine the independent and combined effects of instructional strategies and environmental characteristics.

From an applied perspective, these findings suggest that educators, caregivers, and school administrators should consider integrating diverse types of outdoor activities into early childhood programs. Narrative-based activities may be associated with engagement, attention, and goal-directed behavior, while free play may provide valuable opportunities for the development of coordination and strength through spontaneous exploration [54]. A balanced combination of these approaches may therefore be most effective in supporting different aspects of motor and cognitive development in preschool children.

## 5. Conclusions

The present study explored the associations between different outdoor motor activity approaches and motor competence and inhibitory control in preschool children within a real educational context. The findings suggest that the intervention conditions were associated with different patterns of change across selected motor outcomes.

However, given the quasi-experimental design, baseline differences between groups, the absence of correction for multiple comparisons, and the presence of multiple confounding factors (including environmental context and level of adult involvement), no firm conclusions can be drawn regarding the effectiveness of any specific intervention approach.

Accordingly, the results should be considered preliminary and hypothesis-generating. Future research using randomized controlled designs, standardized environments, and more comprehensive measurement protocols is required to clarify the independent and combined effects of instructional strategies and environmental factors.

## Figures and Tables

**Table 1:** Anthropometric characteristics of the sample.

	N	Gender	Age [Years]	Height [m]	Weight [kg]	Waist [cm]
Storytelling in Motion	36	17m–19f	5.10 ± 0.53	114.1 ± 5.11	21.3 ± 3.32	54.1 ± 3.86
Free Play	22	13m–9f	5.50 ± 0.51	113.3 ± 4.31	19.7 ± 2.56	54.5 ± 2.93
Traditional	29	15m–14f	5.59 ± 0.56	113.6 ± 5.52	20.1 ± 2.85	54.3 ± 3.61
*p*			0.672	0.691	0.673	0.682

**Table 2:** A. Descriptive Statistics (Pre–Post). B. Mixed ANOVA Results.

		(A)		
Variable	Group	N	Pre (M ± SD)	Post (M ± SD)
Heel-to-toe walking	Storytelling	36	49.98 ± 15.78	24.09 ± 18.24
	Free Play	22	39.54 ± 10.62	31.84 ± 15.63
	Traditional	29	35.93 ± 12.73	35.96 ± 14.86
Bicycle Trail	Storytelling	36	2.97 ± 2.44	1.86 ± 1.94
	Free Play	22	1.64 ± 1.87	1.82 ± 2.15
	Traditional	29	1.55 ± 1.38	2.07 ± 1.33
Building Bricks	Storytelling	36	26.46 ± 3.56	25.23 ± 7.71
	Free Play	22	22.02 ± 3.99	24.80 ± 6.31
	Traditional	29	23.02 ± 5.23	28.07 ± 8.99
Medicine Ball	Storytelling	36	181.46 ± 58.72	204.67 ± 42.91
	Free Play	22	175.32 ± 57.99	206.91 ± 49.31
	Traditional	29	173.91 ± 26.22	204.09 ± 56.08
Day/Night	Storytelling	36	4.12 ± 4.70	1.43 ± 2.08
	Free Play	22	2.91 ± 3.34	1.64 ± 2.36
	Traditional	29	5.14 ± 6.01	1.86 ± 2.55
		**(B)**		
**Variable**	**Time *p***	**Time η^2^p**	**Time × Group *p***	**Time × Group η^2^p**
Heel-to-toe walking	<0.001 *	0.216	<0.001 *	0.229
Bicycle Trail	0.414	0.008	<0.001 *	0.194
Building Bricks	0.036 *	0.061	0.042 *	0.085
Platform Bricks (DX)	0.375	0.010	0.002 *	0.145
Medicine Ball	<0.001 *	0.216	0.813	0.005
Day/Night	<0.001 *	0.162	0.425	0.020
Walking/Running Slope	0.008 *	0.112	0.062	0.089
Catching Beanbag	0.005 *	0.045	0.010 *	0.054

* *p* < 0.05.

**Table 3:** Pre–Post Change Scores (Δ) and Best Performing Group.

Variable	Direction	Storytelling Δ	Free Play Δ	Traditional Δ	Best Group
Heel-to-toe walking	↓	−25.89	−7.70	+0.03	Storytelling
Bicycle Trail	↓	−1.11	+0.18	+0.52	Storytelling
Building Bricks	↓	−1.23	+2.78	+5.05	Storytelling *
Platform Bricks (SX)	↓	−1.22	−4.39	+4.83	Free Play
Platform Bricks (DX)	↓	−6.09	−3.48	+5.76	Free Play
Medicine Ball	↑	+23.21	+31.59	+30.18	Free Play
Day/Night	↓	−2.69	−1.27	−3.28	Storytelling
Walking/Running Slope	↓	−1.10	−2.40	+0.10	Free Play

**Note:** ↓ = lower is better; ↑ = higher is better; Δ = Post − Pre. * only group showing improvement.

**Table 4:** ANCOVA Results for Post-Test Outcomes Controlling for Baseline Scores.

Outcome Variable	F (2, 83)	*p*	η^2^p
Heel-to-Toe Walking	4.42	0.015	0.096
Bicycle Trail	5.54	0.006	0.118
Building Bricks	1.13	0.329	0.026
Day/Night Test	0.22	0.806	0.005

*Note: ANCOVA conducted using pre-test scores as covariates.*

**Table 5:** Estimated Marginal Means (Adjusted Means) for Post-Test Scores.

Outcome Variable	Storytelling in Motion	Free Play	Traditional Instruction
Heel-to-Toe Walking	26.10 ± 2.85	30.95 ± 3.12	34.88 ± 3.05
Bicycle Trail	1.72 ± 0.28	1.95 ± 0.31	2.18 ± 0.30
Building Bricks	25.64 ± 1.45	24.98 ± 1.60	26.91 ± 1.55
Day/Night Test	1.55 ± 0.42	1.70 ± 0.48	1.82 ± 0.45

*Values are estimated marginal means ± standard error.*

**Table 6:** Bonferroni Post Hoc Pairwise Comparisons.

Outcome Variable	Comparison	Mean Difference	*p*
Heel-to-Toe Walking	Storytelling vs. Traditional	−8.78	0.009
	Storytelling vs. Free Play	−4.85	0.041
Bicycle Trail	Storytelling vs. Traditional	−0.46	0.004
	Storytelling vs. Free Play	−0.23	0.048

*Only significant comparisons are shown (Bonferroni-adjusted).*

## Data Availability

The data that support the findings of this study are available from the corresponding author (D.D.C.), upon reasonable request.

## Supplementary Materials

The following supporting information can be downloaded at: https://www.mdpi.com/article/10.3390/children13060718/s1, Supplementary File S1 (manuscript-supplementary.pdf).
