# Supplementary material for: Storytelling in Motion: Effects of a Narrative-Based Outdoor Motor Intervention on Motor Competence and Inhibitory Control in Preschool Children—A Quasi-Experimental Study
Source: Children (Basel). 2026 May 22;13(6):718. doi: 10.3390/children13060718 (PMC13296697; doi:10.3390/children13060718)
Supplement: Supplementary file 1 [file children-13-00718-s001.zip › children-4293071-supplementary.pdf]

Outcome Variable	Group	Pre Mean	Pre SD	Post Mean	Post SD
Heel-to-Toe Walking	Storytelling	49.98	15.78	24.09	18.24
	Free Play	39.54	10.62	31.84	15.63
	Traditional	35.93	12.73	35.96	14.86
Bicycle Trail	Storytelling	2.97	2.44	1.86	1.94
	Free Play	1.64	1.87	1.82	2.15
	Traditional	1.55	1.38	2.07	1.33
Building Bricks	Storytelling	26.46	3.56	25.23	7.71
	Free Play	22.02	3.99	24.80	6.31
	Traditional	23.02	5.23	28.07	8.99
Platform Bricks SX	Storytelling	48.06	13.17	46.84	10.54
	Free Play	46.61	8.82	42.22	6.98
	Traditional	43.96	7.11	48.79	7.82
Platform Bricks DX	Storytelling	47.33	15.78	41.24	8.43
	Free Play	41.00	7.76	37.52	3.73
	Traditional	38.40	7.25	44.16	8.83
Medicine Ball	Storytelling	181.46	58.72	204.67	42.91
	Free Play	175.32	57.99	206.91	49.31
	Traditional	173.91	26.22	204.09	56.08
Day/Night	Storytelling	4.12	4.70	1.43	2.08
	Free Play	2.91	3.34	1.64	2.36
	Traditional	5.14	6.01	1.86	2.55
Walking/Running in Slope	Storytelling	10.57	3.16	9.47	2.35
	Free Play	11.59	3.56	9.19	1.69
	Traditional	8.64	2.22	8.74	1.95
